# Triplex viability PCR for simultaneous quantification of *Lactobacillus acidophilus*, *Streptococcus thermophilus*, and *Bifidobacterium bifidum* in live microbial formulations

**DOI:** 10.3389/fmicb.2026.1759060

**Published:** 2026-02-10

**Authors:** Luca Del Pio, Stefania Catone, Giulio Pisani, Giovanna Franciosa

**Affiliations:** 1 National Center for the Control and Evaluation of Medicines, Istituto Superiore di Sanità, Rome, Italy; 2 Department of Biochemistry, Sapienza University, Rome, Italy

**Keywords:** *B. bifidum*, *L. acidophilus*, live biotherapeutic products, multiplex qPCR, probiotics, propidium monoazide, *S. thermophilus*, viability PCR

## Abstract

Live microbial formulations frequently contain strains of *Lactobacillus acidophilus*, *Streptococcus thermophilus* and *Bifidobacterium bifidum* in combination. Accurate determination of microbial identity and viable counts is essential for ensuring product functionality and to meet regulatory requirements. Here, a triplex propidium monoazide (PMA) qPCR assay, referred to as triplex viability PCR (vPCR), was developed for the identification, quantification, and viability assessment of these key microbial species. Primers and TaqMan probes were first evaluated for compatibility and specificity. Heat treatments were applied to inactivate microbial cells, and optimal PMA concentrations were determined to effectively discriminate between live and dead microbial cells. Amplification conditions were optimized to enable the simultaneous generation of standard curves for all three target species using their respective primer/probe sets. The triplex vPCR assay produced three distinct amplification signals and the corresponding standard curves demonstrated good assay reproducibility, with R^2^ values greater than 0.98 and reaction efficiencies between 90 and 110%. The optimized protocol allowed accurate viable quantification of *L. acidophilus* and *B. bifidum* over a range of approximately 10 to 10^7^ CFU/mL, and of *S. thermophilus* over 30 to 3×10^7^ CFU/ml. Application of the protocol to a commercial multi-species live microbial formulation provided consistent viable quantification for all three species. Plate counts were generally lower than both triplex qPCR and triplex vPCR measurements, while triplex vPCR values were lower than triplex qPCR, reflecting selective exclusion of DNA from dead cells. By combining multiplexing, high-efficiency reagents, and a PMA pretreatment that selectively prevents amplification from non-viable cells, this triplex vPCR method overcomes the limitations of culture-dependents techniques and standard qPCR, improves analytical throughput, reduces variability, ensuring accurate quantification of viable microorganisms.

## 1 Introduction

Over the past decades, knowledge about the beneficial effects of specific live microbial strains and their applications across different health conditions has grown considerably, along with the understanding that the bacteria must be alive and administered in sufficient quantities to confer the intended health benefits. At the same time, the range of products containing high levels of live microorganisms, including probiotic dietary supplements and live biotherapeutic products (LBPs), has expanded, highlighting the need for accurate and efficient quality control (O’Toole et al., 2017; Forssten et al., 2020; McChalicher and Auniņš, 2022; Campaniello et al., 2023).

These formulations often contain multiple microbial species. An accurate assessment of the identity, number and viability of each microbial species is essential to verify label claims and ensure consistent product quality, as required for LBPs and recommended for probiotic supplements (FAO/WHO, 2002; International Probiotics Association, 2017; European Pharmacopoeia Commission, 2019).

Viability and quantification of microorganisms in products are traditionally evaluated using culture-based methods, such as plate counting. Identification typically relies on selective media and may require additional biochemical or molecular characterization. Although widely accepted and still considered the gold standard, these methods have several well-known limitations, including long incubation times, labor-intensive procedures, and reduced sensitivity in detecting stressed or slow-growing cells, such as viable but non-culturable (VBNC) cells (Boyte et al., 2023; Shehata et al., 2025).

Moreover, they often lack the specificity needed to discriminate among species with similar growth requirements and phenotypic characteristics. For this reason, several alternative methods have been developed to assess the quality of live microbial products. These include nucleic acid-based approaches (e.g., PCR, genome sequencing, and hybridization techniques), immunological and cell-based assays (e.g., ELISA and flow cytometry) and biophysical methods (e.g., spectroscopy and mass spectrometry) (Zielińska et al., 2018; Boyte et al., 2023; Shehata et al., 2025; Sun et al., 2025).

However, most of these approaches cannot simultaneously assess identity, viability and quantity in a single workflow, necessitating the combined use of multiple techniques, thus increasing costs, complexity and variability (Sun et al., 2025). Propidium monoazide (PMA) quantitative PCR (qPCR), also referred to as viability-PCR (vPCR), has emerged as a promising approach to overcome these limitations (Chen et al., 2022; Wendel, 2022). PMA selectively binds to the DNA of membrane-compromised (dead or severely injured) cells and, upon light activation, prevents its subsequent amplification, thereby enabling the selective detection of intact and viable cells (Nocker et al., 2007). In this context, we recently developed a vPCR assay for detection of *Lactobacillus acidophilus* and *Bifidobacterium bifidum* in separate reactions (Catone et al., 2024).

The present study advances our previous approach by expanding the method from a single-target vPCR to a multiplex vPCR format and by extending the assay to include a third microbial species (*Streptococcus thermophilus*). The optimized triplex assay was applied to a commercial multi-species live microbial formulation, demonstrating its efficiency and practical relevance for quantitative viability assessment and quality control.

To the best of our knowledge, methods for the simultaneous identification of multiple viable bacterial species in probiotic and LBP preparations are currently lacking; therefore, the approach described here represents a rapid, sensitive, and cost-effective alternative.

## 2 Materials and methods

### 2.1 Microbial strains and growth conditions

The microorganisms used in this study included three “reference” strains of the target species, i.e., *L. acidophilus* DSM 20079, *S. thermophilus* DSM 20617, and *B. bifidum* DSM 20456; and eight strains from non-target species, including *L. delbruecki* subsp. *bulgaricus* DSM 20081, *L. delbruecki* subsp. *lactis* DSM 20072, *B. breve* DSM 20213, *B. animalis* subsp. *lactis* DSM 10140, *Lactiplantibacillus plantarum* DSM 20174, *Lacticaseibacillus paracasei* DSM 5622, *Bacillus clausii* DSM 8716, and *Enterococcus faecium* SF68. All strains were obtained from the German Collection of Microorganisms and Cell Cultures (DSMZ Braunschweig, Germany), except for *E. faecium* SF68 which was sourced from the Istituto Superiore di Sanità culture collection (Rome, Italy). Their identity was confirmed by 16S rDNA sequencing, as described by Boye et al. (1999). Strains were stored at – 80 °C in microbank cryogenic vials (Prolab Diagnostics, Richmond Hill, ON, Canada) until use.

*S. thermophilus* was routinely cultured in tryptone soy broth or agar (TSB/TSA) (Oxoid, ThermoFisher Scientific, Basingstoke, UK) under aerobic conditions at 37 °C for 24 h. Lactobacilli, bifidobacteria and enterococci strains were grown in de Man, Rogosa, Sharpe broth or agar (MRSB/MRSA) (Oxoid) supplemented with 0.05% L-cysteine HCl when required, and incubated anaerobically at 37 °C for 24–48 h. Anaerobic jars and gas generating kits (Oxoid) were used to create the anaerobic conditions. *B. clausii* was cultured on nutrient agar (pH ⁓ 9) and incubated aerobically at 37 °C for 48 h.

### 2.2 Preparation of heat-killed suspensions

Heat-killed suspensions of *L. acidophilus* DSM 20079 and *B. bifidum* DSM 20456 were prepared by heating at 100 °C for 10 min, as previously described (Catone et al., 2024). These conditions did not fully inactivate *S. thermophilus* DSM 20617. Therefore, 1 mL aliquots of overnight broth cultures of this strain were centrifuged, washed with 0.9% NaCl, and resuspended in the same saline solution to an optical density at 600 nm (OD600) of approximately 1; the resulting suspensions were then subjected to heat treatments at 100 °C for 15, 20, 22, 25 and 30 min. Lethality was confirmed by plating on TSA. All experiments were performed in duplicate.

### 2.3 Enumeration by plate count

Serial dilutions of overnight cell suspensions of the three target strains, previously adjusted to an OD600 ⁓ 1, were spread on selective agar media. Specifically, *S. thermophilus* was enumerated on M17 agar (Biolife, Milan, Italy), *L. acidophilus* on MRSA supplemented with 0.002% bromophenol blue (Aureli et al., 2008), and *B. bifidum* on Bifidus Selective Medium agar (Sigma-Aldrich, St. Louis, Missouri). The plates were then incubated at 37 °C for 48–72 h under aerobic or anaerobic conditions, according to the target strain requirements. Subsequently, plates containing 30–300 presumptive colonies were used to enumerate the microorganisms. Results were expressed as colony-forming units (CFU) per mL of broth culture. For enumeration of the targeted species in live microbial formulations, experiments were carried out in triplicate for each target strain, and data were recorded as mean ± standard deviation.

### 2.4 DNA extraction

For specificity verification, DNA was extracted from pure broth cultures of all strains listed above using a Mag-Bind cfDNA kit (Omega Bio-Tek, Norcross, GA, USA).

To establish standard curves for the target strains, genomic DNA was extracted from pure broth cultures of the reference strains using the DNeasy Tissue kit (Qiagen, Hilden, Germany), following the manufacturer’s instructions. The quantity and quality of extracted DNA were assessed using an ultraviolet spectrophotometer (Biophotometer, Eppendorf, Milan, Italy). DNA samples were stored at – 20 °C until use.

### 2.5 Primers and TaqMan probes

The sequences of the primers and TaqMan probes used in this study to simultaneously detect *L. acidophilus*, *S. thermophilus* and *B. bifidum*, are shown in Table 1. The fluorescent labels of the three species-specific probes were chosen to allow simultaneous multiplex detection, with ROX dye used as the passive reference. Primers and TaqMan probes were synthesized by Integrated DNA Technologies (IDT) (Leuven, Belgium).

**Table 1:** Primers and probes used in this study.

Primer/probe set	Sequence	Modification^c^	Target gene
*L. acidophilus^a^*			
La forward primer	5′- GAA AGA GCC CAA ACC AAG TGA TT - 3′		
La probe	5′- TAC CAC TTT GCA GTC CTA CA - 3′	5’Cy5/TAO/IBRQ 3′	Intergenic spacer region
La reverse primer	5′- CTT CCC AGA TAA TTC AAC TAT CGC TTA- 3’		
*B. bifidum^b^*			
Bb forward primer	5′- ACC GAA TTC GCC TGT CAC TT - 3′		
Bb probe	5′- CCG CTG GAT GTG AAC - 3′	5’ FAM/BHQ1 3’	ATP-binding protein gene
Bb reverse primer	5′- ACG GCG CGG ATT CGT − 3′		
*S. thermophilus^c^*			
St forward primer	5′- TGA AGC TTT AGC GAC ACT AGT AA - 3’		
St probe	5′- TCC TCG CTT TGG ACT TGG TCA TGG - 3’	5’ SUN/ZEN/IBFQ 3’	ATPase V gene
St reverse primer	5′- GCA GAG ACA GCT AGA AAG AGA C - 3’		

^a^Haarman and Knol (2006). ^b^Singh et al. (2013). ^c^This study.

Specifically, the primers and TaqMan probes used for *L. acidophilus* and *B. bifidum* were the same as those used in our previous work, except for the Cy5 fluorophore of the *L. acidophilus* probe that replaced the previously used FAM fluorophore (Catone et al., 2024).

In contrast, primers and TaqMan probes specific for *S. thermophilus* were newly designed using the online tools available on the IDT website (https://eu.idtdna.com/Primerquest/Home/Index). The designed sequences were subsequently evaluated to exclude potential heterodimer formation with the primer/probe sets for *L. acidophilus* and *B. bifidum*. The sequences targeted the *S. thermophilus* species-specific *ATPase* V gene (GenBank accession no CP019935.1) (Stachelska et al., 2024). Their specificity was assessed *in silico* by Basic Local Alignment Search Tool (BLAST) analysis using nucleotide tool on standard database nucleotide collection (nr/nt) (NCBI), and *in vitro* by singleplex qPCR using the *S. thermophilus* primer/probe set with genomic DNA from all strains listed above.

### 2.6 Triplex qPCR assay

The triplex qPCR assay was performed using the Applied Biosystems (AB) 7,500 Real-Time PCR System (Waltham, MA, USA) with software version 2.3. The cycling conditions consisted of an initial denaturation at 95 °C for 2 min, followed by 45 cycles of denaturation at 95 °C for 10 s and annealing/extension at 60 °C for 30 s. Each reaction (20 μL final volume) consisted of 1X SensiFast Lo-ROX Master Mix (Aurogene, Rome, Italy), 250 nM of each primer, 250 nM of each TaqMan probe and 6 μL of template DNA. To verify the absence of contamination and non-specific amplification, blank controls were prepared by replacing DNA with DNAse/RNAse-free water (Bioline, London, UK). All triplex qPCR assays were run in duplicate.

### 2.7 Creation of standard curves

To quantify *L. acidophilus*, *S. thermophilus* and *B. bifidum* in test samples, the cycle threshold (Ct) values obtained by triplex qPCR were interpolated from standard calibration curves. These curves were prepared from 10-fold serial dilutions of genomic DNA extracted from broth cultures of *L. acidophilus* DSM 20079, *S. thermophilus* DSM 20617, and *B. bifidum* DSM 20456, for which the viable cell counts were determined by the plate count method and expressed as CFU/ml. The dilution series used to generate the standard curves comprised at least seven concentration levels, corresponding to approximately 10–10^7^ CFU/mL. Genomic DNA stocks were divided into single-use aliquots and stored at −20 °C to prevent repeated freeze–thaw cycles prior to standard curve construction. The experiments were performed three times.

### 2.8 Optimization of PMA treatment

The PMA treatment used to differentiate viable from dead *S. thermophilus* cells was optimized following a procedure adapted from that previously applied to *L. acidophilus* and *B. bifidum* (Catone et al., 2024). Briefly, overnight broth cultures of the three target microorganisms were adjusted to OD600 ~ 1 and divided into live and heat-inactivated aliquots, with loss of viability verified by plate count. PMA (Biotium, Fremont, CA, USA) at final concentrations of 25, 50 and 100 μM was added to 250 μL aliquots and incubated in the dark for 10 min. All samples were placed on ice and photoactivated for 5 min using a 500 W halogen light source at 20 cm distance, under gentle agitation.

DNA was then extracted and analyzed by singleplex qPCR with the species- specific primer/probe sets (two replicates). PMA cytotoxicity was evaluated by comparing plate counts of treated versus untreated live cells. The assay was conducted twice.

To verify the optimal PMA concentration, cell mixtures were prepared by combining 250 μL of viable cells with 250 μL of heat-inactivated cells; live and dead cells alone served as controls. Mixtures and controls were treated with the optimal PMA concentration as determined above, and processed in duplicates. DNA was then extracted and analyzed by qPCR. The experiment was performed twice.

### 2.9 Application of the triplex vPCR assay for quantifying the targeted species in live microbial formulations

A commercial live microbial capsule products containing the three target species at labeled levels of ⁓ 10^9^ CFU/capsule were purchased, stored at 4 °C, and analyzed before their expiration dates. Three capsules from the product were examined. Briefly, each capsule was suspended in 10 mL of sterile saline. Aliquots (1 mL) of each suspension were treated with 25 μM PMA and photoactivated at the conditions described above. DNA was extracted from both PMA-treated and untreated suspensions using the DNeasy Blood and Tissue kit (Qiagen), diluted 1:100, and analyzed by triplex qPCR using *L. acidophilus*, *S. thermophilus* and *B. bifidum* specific primer/probe sets. Each reaction was performed in duplicate, with no-template controls included in each run. Serial dilutions of DNA from the three reference strains were prepared and analyzed in parallel (in duplicate) to generate standard curves. Concentrations of each species in the samples were calculated by interpolation against the respective standard curve, with slope and linear correlation automatically determined by the AB 7500 system software. Results were compared with those obtained by plate count as above described.

### 2.10 Statistical analysis

Comparisons among analytical methods were assessed using Student’s t-test for unpaired data (as the measurements were obtained from independent samples), and a threshold of *p < 0.05* was adopted to denote statistically significant differences. Statistical analyses were conducted using GraphPad Prism version 10.5.0 (San Diego, CA, USA).

## 3 Results

### 3.1 Optimization of triplex vPCR

The specificity of the primer/probe sets for *L. acidophilus* and *B. bifidum* had been previously established (Catone et al., 2024). The newly designed *S. thermophilus* primer/probe set was evaluated in this study and confirmed to be specific by both *in silico* analysis and *in vitro* testing (data not shown).

**Figure 1:**
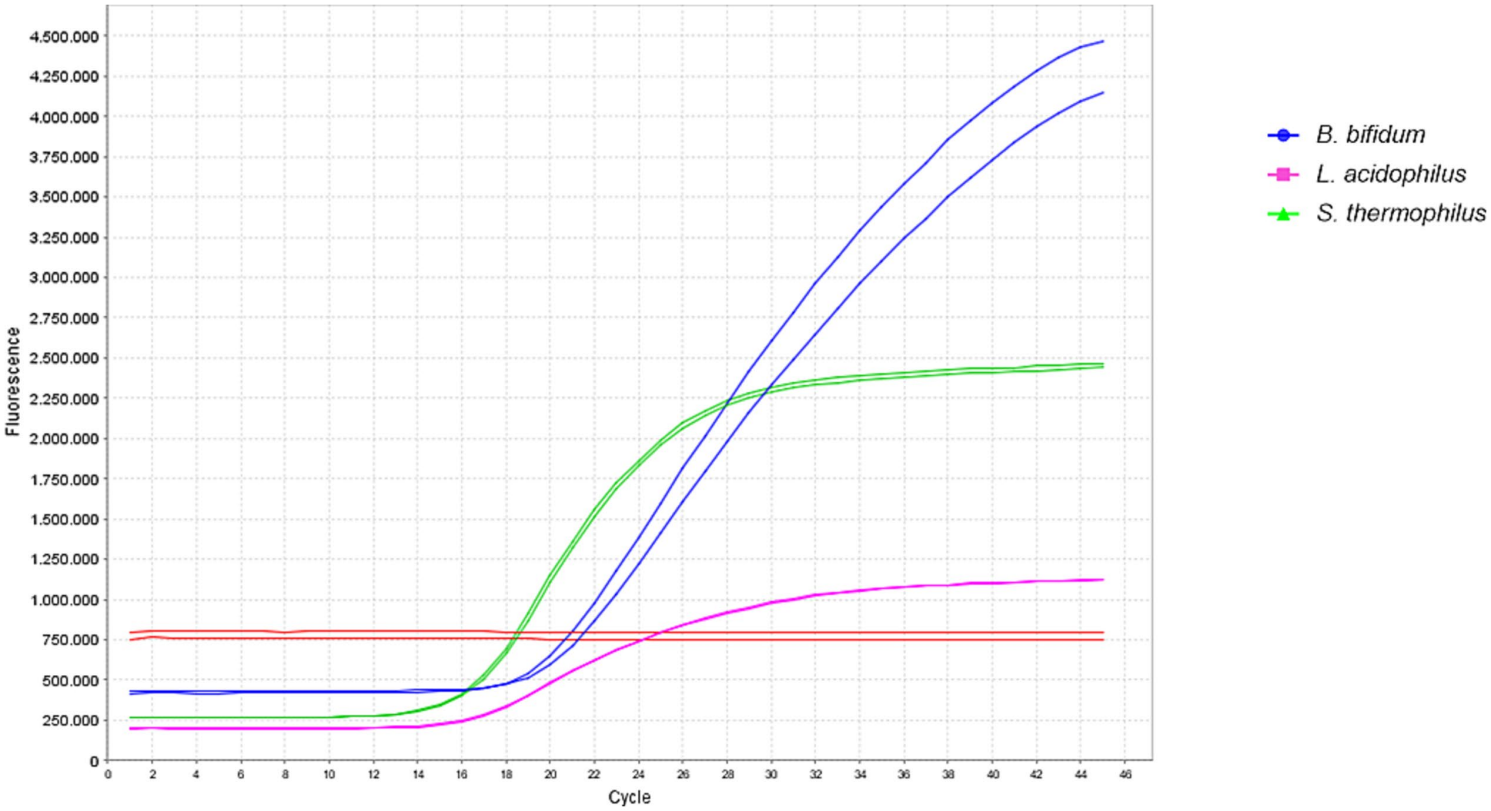
Amplification curves obtained in duplicate for the three primer/probe sets targeting *L. acidophilus*, *S. thermophilus,* and *B. bifidum*. Horizontal lines indicate the threshold fluorescence value, with ROX used as the passive reporter dye.

The triplex qPCR generated three distinct amplification curves with strong fluorescence signals, demonstrating that the primer/probe sets and applied conditions efficiently and accurately amplified their respective targets in a single reaction (Figure 1).

**Figure 2:**
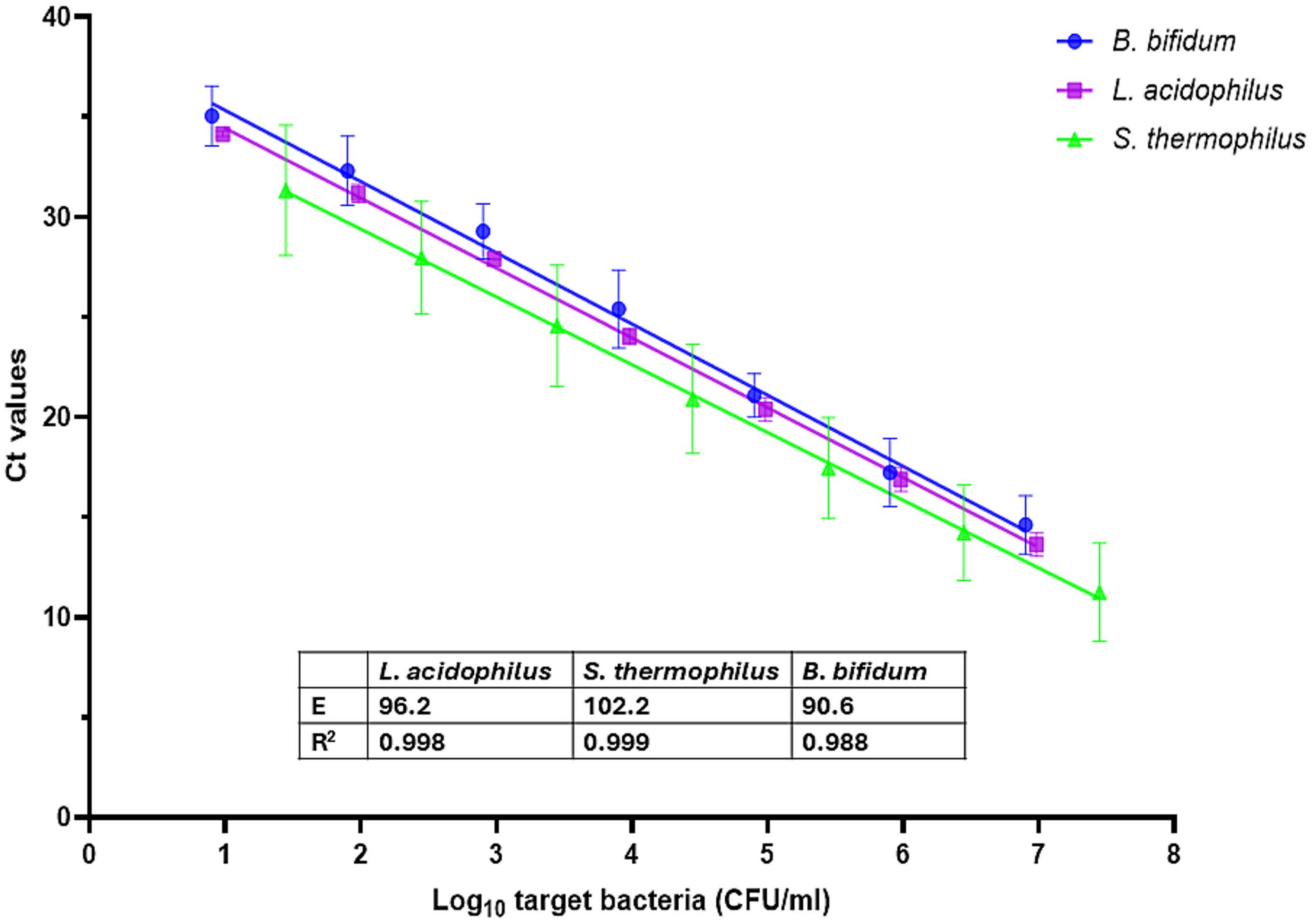
Standard curves and sensitivity of the triplex qPCR for the different microbial species. Each point represents the mean cycle thresholds (Ct) values of three independent experiments performed in duplicate, with error bars showing the standard deviation. E: Efficiency. R^2^: Coefficient of determination.

Following DNA extraction, the average DNA yields were 92 ng/μl for *L. acidophilus*, 60 ng/μl for *S. thermophilus*, and 80 ng/μl for *B. bifidum*. Standard curves obtained under triplex qPCR conditions for the three target microorganisms are shown in Figure 2. Assay reproducibility, as indicated by linear correlation (R^2^), and reaction efficiency (E), were within acceptable range of values, i.e., R^2^ > 0.98 and E between 90 and 110% (Kralik and Ricchi, 2017) (Figure 2).

**Figure 3:**
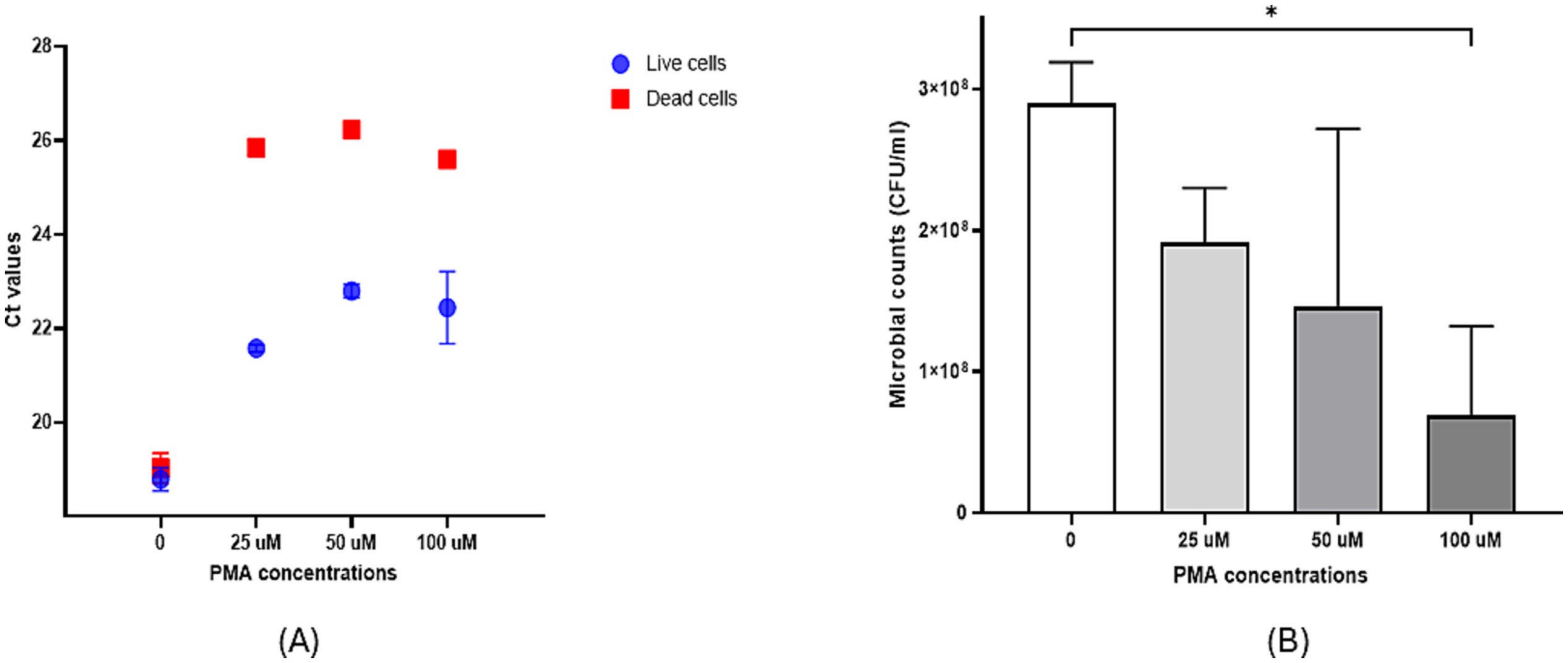
**(A)** Effects of pretreatment with different concentrations of PMA on Ct values of live and dead *S. thermophilus* cells after qPCR. **(B)** Cytotoxic effects of different PMA concentrations on *S. thermophilus* cells. *p < 0.05.

A heat treatment of 30 min at 100 °C was necessary to inactivate *S. thermophilus* cells. Consistent with our findings for *L. acidophilus* and *B. bifidum* (Catone et al., 2024), a PMA concentration of 25 μM was optimal for inhibiting DNA amplification from dead *S. thermophilus* cells while minimally affecting amplification from viable cells. In fact, the 25 μM concentration caused the smallest Ct variation in live cells compared with the other PMA concentrations tested, while still producing a marked Ct increase in dead cells (Figure 3A). Moreover, while the 100 μM PMA concentration significantly reduced microbial counts (*p < 0.05*) indicating strong cytotoxicity, the 50 μM and 25 μM concentrations did not, with 25 μM showing the lowest cytotoxic effect in live *S. thermophilus* cells (Figure 3B).

**Figure 4:**
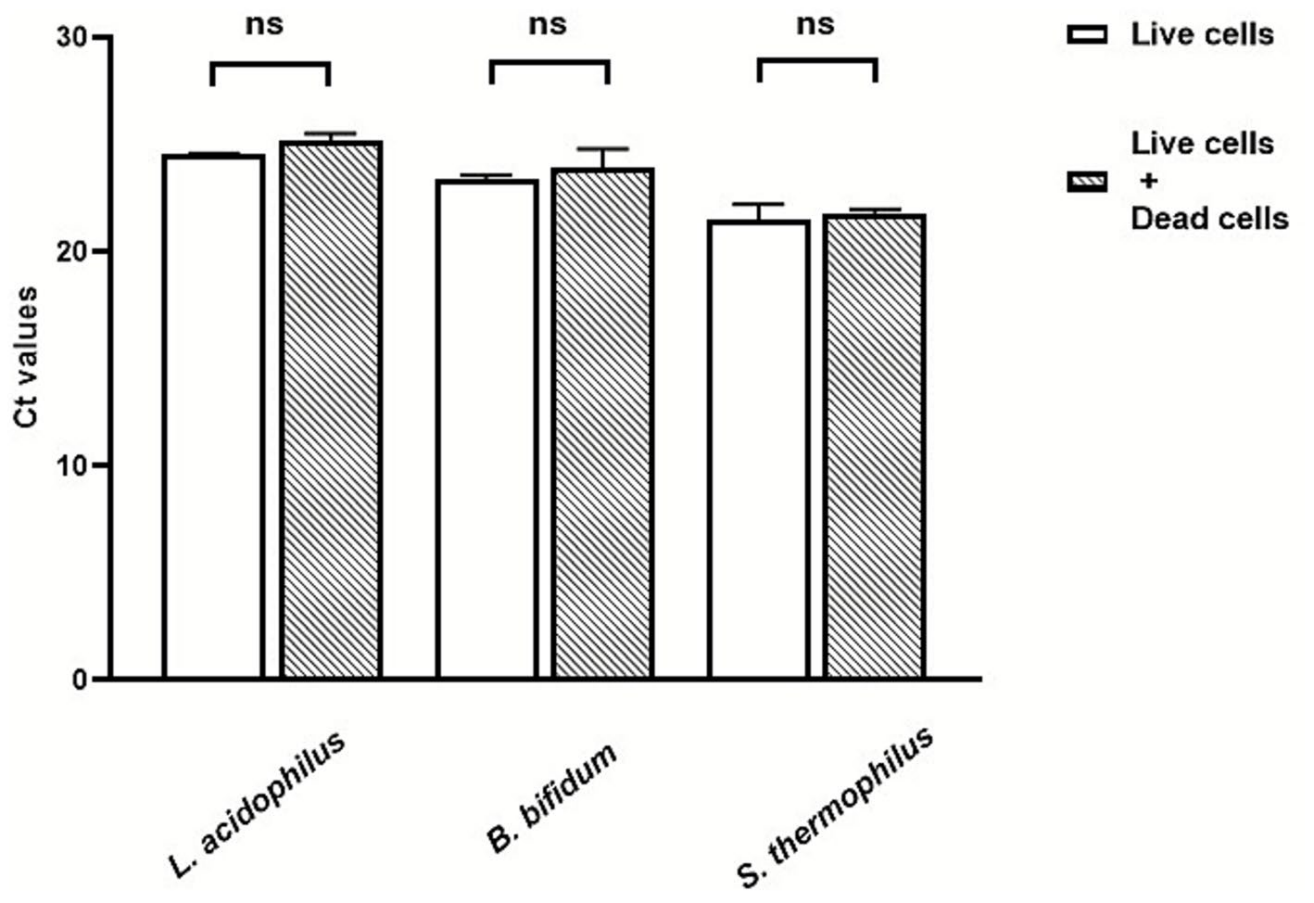
Effects of pretreatment with 25 μM PMA on Ct values of live cells of *L. acidophilus* and *S. thermophilus* (⁓ 10^8^ CFU/mL) and *B. bifidum* (⁓ 10^7^ CFU/mL), tested either alone or in the presence of an equal amount of dead cells. NS: No significant differences (*p* > 0.05).

When aliquots of live cells (⁓ 10^7^ or 10^8^ CFU/mL, depending on the target microorganism) were treated with 25 μM PMA, either alone or mixed with equal amounts of dead cells at the same concentrations, no significant differences in Ct values were observed between the two groups for any of the target microorganisms (Figure 4). This result confirmed that the selected PMA treatment conditions efficiently inhibited amplification from dead cells while allowing selective quantification of viable cells. Under these conditions, the assay limits of detection were approximately 10 CFU/mL for *L. acidophilus* and *B. bifidum* and 30 CFU/mL for *S. thermophilus.*

### 3.2 Analyses of a multispecies live microbial formulation

**Figure 5:**
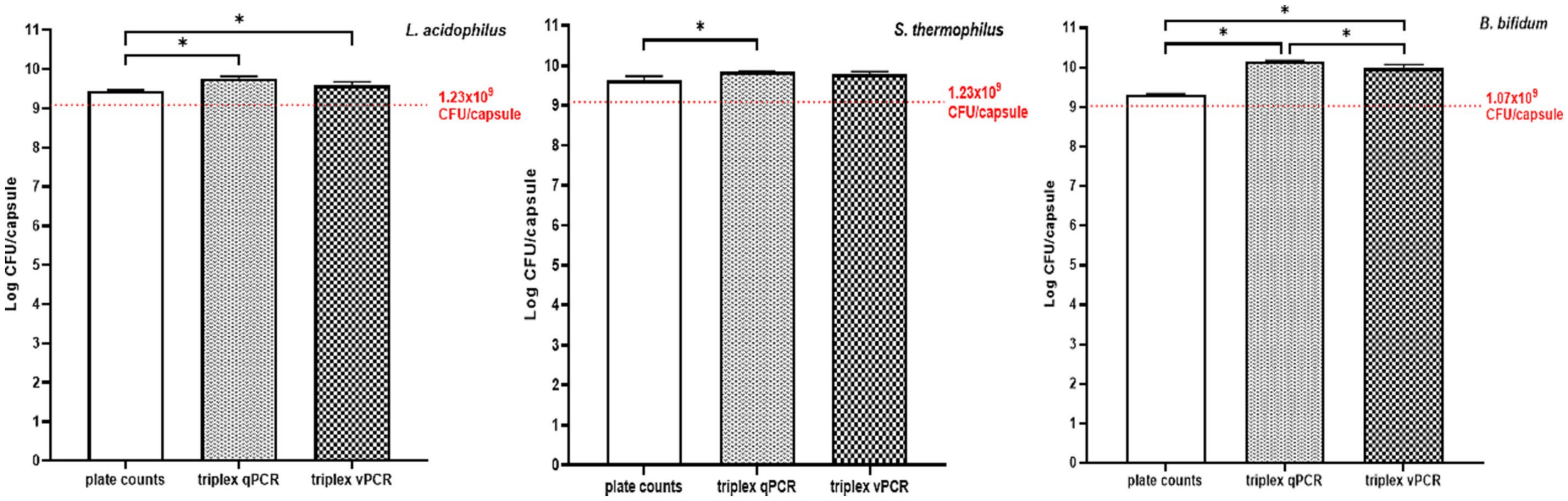
Quantities of *L. acidophilus*, *S. thermophilus,* and *B. bifidum* in a commercial multispecies live microbial formulation as determined by plate count, triplex qPCR, and triplex vPCR. Red lines indicate the labeled quantities. Bars represent the mean values of three independent analyses performed in duplicate, while error bars indicate the corresponding standard deviation. Statistically significant differences between analytical methods, as assessed by Student’s *t*-test, are indicated by * when *p < 0.05*, and are shown between the corresponding bars.

Figure 5 shows the quantities of *L. acidophilus*, *S. thermophilus* and *B. bifidum* in a multispecies live microbial formulation, as determined by plate counts, triplex qPCR, and triplex vPCR.

Plate counts of the three target microorganisms were significantly lower than the quantities determined by both triplex qPCR and triplex vPCR methods, except for *S. thermophilus* for which plate counts, although lower, did not differ significantly from the triplex vPCR results (Figure 5). As expected, triplex vPCR values were generally lower than those obtained by triplex qPCR, reflecting the selective exclusion of DNA from dead cells by PMA treatment; however, the difference was statistically significant only for *B. bifidum*.

Overall, the quantities of the three microorganisms determined in the product by plate counts, triplex qPCR and triplex vPCR were all significantly higher than the amounts stated on the product label (Figure 5).

## 4 Discussion

This work describes a novel triplex-qPCR method coupled with PMA pretreatment (triplex vPCR) enabling the simultaneous viability quantification of *L. acidophilus*, *S. thermophilus*, and *B. bifidum* with high specificity, sensitivity and accuracy. Despite differences in absolute DNA yield among bacterial species, the strong linear correlation observed between Ct values and bacterial counts (R^2^ > 0.98) indicates that extraction yield variability did not significantly affect assay linearity or quantitative performance.

Singleplex qPCR coupled with PMA treatment has been applied for the quantification of viable *L. gasseri*, *L. salivarius*, *L. acidophilus*, and *B. bifidum* in probiotic products and LBPs (Lai et al., 2017; Catone et al., 2024; Guo et al., 2024), as well as for *S. thermophilus*, and several lactic acid bacteria, including *L. paracasei*, *L. delbruecki*, *L. helveticus*, *L. plantarum, Lacticaseibacillus rhamnosus*, and *Lactococcus lactis* in yogurt and other fermented milk products (García-Cayuela et al., 2009; Scariot et al., 2018; Yang et al., 2021; Shi et al., 2022; Marole et al., 2024).

In contrast, to the best of our knowledge, multiplex qPCR assays for the analysis of multispecies microbial formulations have not yet been reported.

In the present assay, the three primer/probe sets, each labeled with a distinct fluorophore, were successfully combined in a single reaction, showing high specificity for their respective target microorganisms and no cross-reactivity either among the targets or with non-target microorganisms.

The implementation of multiplexing, together with the use of a high-efficiency master mix, further improved analytical throughput by reducing both assay time and reagent consumption.

In addition, pretreatment with 25 μM PMA effectively prevented DNA amplification from dead microbial cells, allowing selective quantification of viable cells while inducing the lowest cytotoxic effects on live cells. This PMA concentration is lower than the 50 μM and 100 μM concentrations used by other authors in combination with singleplex qPCR for viable probiotic detection (García-Cayuela et al., 2009; Lai et al., 2017; Scariot et al., 2018; Yang et al., 2021; Shi et al., 2022; Guo et al., 2024; Marole et al., 2024); however, PMA-induced cytotoxicity was not preliminary evaluated in most of those studies, which may have led to underestimation of viable cell numbers.

Overall, these improvements greatly enhance the method’s practical applicability for quality control of multispecies formulations, supporting accurate measurements and consumer confidence.

When applied to a marketed formulation, quantitative results generated by the triplex vPCR assay were consistently higher than plate count values and lower than those obtained by triplex qPCR for all three target microorganisms. This highlights the advantages of the method, as it overcomes the intrinsic limitations of conventional approaches: plate counts underestimate microbial numbers by failing to detect physiologically stressed or VBNC cells, whereas standard qPCR overestimates counts by amplifying DNA from both live and dead cells. By selectively excluding DNA from non-viable cells, the triplex vPCR allows a more accurate assessment of total viable beneficial bacteria, including VBNC cells that may remain metabolically active and resuscitate under favorable conditions (Oliver, 2005).

Notably, quantities measured by plate counts, triplex qPCR and triplex vPCR were all significantly higher than the declared amounts for the three microorganisms. This suggests that surplus cells may have been included in the product, which is an established practice to ensure that the labeled specifications are met through its shelf life (Fiore et al., 2020). In this context, the detection limits of the triplex vPCR assay were well suited for the analysis of multispecies microbial formulations, which typically contain more than 10^6^–10^7^ CFU / g of live microorganisms to ensure an effective daily intake (Marco et al., 2020; Boyte et al., 2023).

Since the method was developed for live microbial formulations, the impact of different sample matrices on its performance remains to be investigated, and further studies are required to fully assess its robustness and suitability for different product types.

Finally, while the assay reliably distinguishes viable cells of each target species, which is advantageous when products contain a single strain per species, differentiating individual strains within the same species would require primer/probe sets targeting strain-specific genetic markers, an information that is often unavailable or insufficiently characterized.

## Data Availability

The raw data supporting the conclusions of this article will be made available by the authors, without undue reservation.

## References

[ref1] Aureli P. Fiore A. Scalfaro C. Franciosa G. (2008). Microbiological and molecular methods for analysis of probiotic-based food supplements for human consumption. Rapp. Istisan 8:63.

[ref2] Boye K. Høgdall E. Borre M. (1999). Identification of bacteria usingtwo degenerate 16S rDNA sequencing primers. Microbiol. Res. 154, 23–26. doi: 10.1016/S0944-5013(99)80030-5, 10356793

[ref3] Boyte M. E. Benkowski A. Pane M. Shehata H. R. (2023). Probiotic and postbiotic analytical methods: a perspective of available enumeration techniques. Front. Microbiol. 14:1304621. doi: 10.3389/fmicb.2023.1304621, 38192285 PMC10773886

[ref4] Campaniello D. Bevilacqua A. Speranza B. Racioppo A. Sinigaglia M. Corbo M. R. (2023). A narrative review on the use of probiotics in several diseases: evidence and perspectives. Front. Nutr. 10:1209238. doi: 10.3389/fnut.2023.1209238, 37497058 PMC10368401

[ref5] Catone S. Iannantuono S. Genovese D. Von Hunolstein C. Franciosa G. (2024). Viability-PCR for the selective detection of *Lactobacillus acidophilus* and *Bifidobacterium bifidum* in live bacteria-containing products. Front. Microbiol. 15:1400529. doi: 10.3389/fmicb.2024.1400529, 39021625 PMC11251893

[ref6] Chen M. Lan X. Zhu L. Ru P. Xu W. Liu H. (2022). PCR-mediated nucleic acid molecular recognition technology for detection of viable and dead foodborne pathogens. Foods 11:2675. doi: 10.3390/foods11172675, 36076861 PMC9455676

[ref7] European Pharmacopoeia Commission (2019). General monograph on live biotherapeutic products for human use (3053). Eur. Pharm. 9, 6522–6523.

[ref8] FAO/WHO (2002). Guidelines for the evaluation of probiotics in food. Rome: FAO.

[ref9] Fiore W. Arioli S. Guglielmetti S. (2020). The neglected microbial components of commercial probiotic formulations. Microorganisms 8:1177. doi: 10.3390/microorganisms8081177, 32756409 PMC7464440

[ref10] Forssten S. D. Laitila A. Makonnen J. Ouwehand A. C. (2020). Probiotic triangle of success: strain production, clinical studies and product development. FEMS Microbiol. Lett. 367:fnaa167. doi: 10.1093/femsle/fnaa167, 33049046 PMC7578568

[ref11] García-Cayuela T. Tabasco R. Peláez C. Requena T. (2009). Simultaneous detection and enumeration of viable lactic acid bacteria and bifidobacteria in fermented milk by using propidium monoazide and real-time PCR. Int. Dairy J. 19, 405–409. doi: 10.1016/j.idairyj.2009.02.001

[ref12] Guo L. Ze X. Feng H. Liu Y. Ge Y. Zhao X. . (2024). Identification and quantification of viable *Lacticaseibacillus rhamnosus* in probiotics using validated PMA-qPCR method. Front. Microbiol. 15:1341884. doi: 10.3389/fmicb.2024.1341884, 38298895 PMC10828034

[ref13] Haarman M. Knol J. (2006). Quantitative real-time PCR analysis of fecal *Lactobacillus* species in infants receiving a prebiotic infant formula. Appl. Environ. Microbiol. 72, 2359–2365. doi: 10.1128/AEM.72.4.2359-2365.2006., 16597930 PMC1448991

[ref14] International Probiotics Association (2017). Best practices guidelines for probiotics. Québec, Canada: International Probiotics Association. Available online at: https://www.crnusa.org/sites/default/files/pdfs/CRN-IPA-Best-Practices-Guidelines-for-Probiotics.pdf

[ref15] Kralik P. Ricchi M. (2017). A basic guide to real time PCR in microbial diagnostics: definitions, parameters, and everything. Front. Microbiol. 8:108. doi: 10.3389/fmicb.2017.00108. 28210243 PMC5288344

[ref16] Lai C. Wu S. Pang J. Ramireddy L. Chiang Y. Lin C. . (2017). Designing primers and evaluation of the efficiency of propidium monoazide - quantitative polymerase chain reaction for counting the viable cells of *Lactobacillus gasseri* and *Lactobacillus salivarius*. J. Food Drug Anal. 25, 533–542. doi: 10.1016/j.jfda.2016.10.004, 28911639 PMC9328820

[ref17] Marco M. L. Hill C. Hutkins R. Slavin J. Tancredi D. J. Merenstein D. . (2020). Should there be a recommended daily intake of microbes? J. Nutr. 150, 3061–3067. doi: 10.1093/jn/nxaa323, 33269394 PMC7726123

[ref18] Marole T. A. Sibanda T. Buys E. M. (2024). Assessing probiotic viability in mixed species yogurt using a novel propidium monoazide (PMAxx)-quantitative PCR method. Front. Microbiol. 15:1325268. doi: 10.3389/fmicb.2024.1325268, 38389538 PMC10882272

[ref19] McChalicher C. W. Auniņš J. G. (2022). Drugging the microbiome and bacterial live biotherapeutic consortium production. Curr. Opin. Biotechnol. 78:102801. doi: 10.1016/j.copbio.2022.102801, 36228531

[ref20] Nocker A. Sossa-Fernandez P. Burr M. D. Camper A. K. (2007). Use of propidium monoazide for live/dead distinction in microbial ecology. Appl. Environ. Microbiol. 73, 5111–5117. doi: 10.1128/AEM.02987-06. 17586667 PMC1951001

[ref21] O’Toole P. W. Marchesi J. R. Hill C. (2017). Next-generation probiotics: the spectrum from probiotics to live biotherapeutics. Nat. Microbiol. 2:17057. doi: 10.1038/nmicrobiol.2017.57 28440276

[ref22] Oliver J. D. (2005). The viable but nonculturable state in bacteria. J. Microbiol. 43, 93–100. 15765062

[ref23] Scariot M. C. Venturelli G. L. Prudêncio E. S. Arisi A. C. M. (2018). Quantification of *Lactobacillus paracasei* viable cells in probiotic yoghurt by propidium monoazide combined with quantitative PCR. Int. J. Food Microbiol. 264, 1–7. doi: 10.1016/j.ijfoodmicro.2017.10.021, 29073460

[ref24] Shehata H. R. Pane M. Buys E. M. Koshy B. Vegge C. S. Schoeni J. L. (2025). Editorial: emerging technologies for viability enumeration of live microorganisms. Front. Microbiol. 15:1546438. doi: 10.3389/fmicb.2024.1546438, 39886210 PMC11780490

[ref25] Shi Z. Li X. Fan X. Xu J. Liu Q. Wu Z. . (2022). PMA-qPCR method for the selective quantitation of viable lactic acid bacteria in fermented milk. Front. Microbiol. 13:984506. doi: 10.3389/fmicb.2022.984506, 36160254 PMC9491339

[ref26] Singh N. Arioli S. Wang A. Villa C. R. Jahani R. Song Y. S. . (2013). Impact of *Bifidobacterium bifidum* MIMBb75 on mouse intestinal microorganisms. FEMS Microbiol. Ecol. 85, 369–375. doi: 10.1111/1574-6941.12124. 23551062

[ref27] Stachelska M. A. Ekielski A. Karpiński P. Żelaziński T. Kruszewski B. (2024). New genetic determinants for qPCR identification and enumeration of selected lactic acid bacteria in raw-milk cheese. Molecules 29:1533. doi: 10.3390/molecules29071533, 38611811 PMC11013805

[ref28] Sun X. Zhang J. Chen J. Yang L. Pang X. Fan Y. . (2025). Conventional versus emerging techniques in probiotic enumeration: a comprehensive review. Crit. Rev. Food Sci. Nutr. 66, 541–564. doi: 10.1080/10408398.2025.2527355, 40608865

[ref29] Wendel U. (2022). Assessing viability and stress tolerance of probiotics—a review. Front. Microbiol. 12:818468. doi: 10.3389/fmicb.2021.818468, 35154042 PMC8829321

[ref30] Yang Y. Liu Y. Shu Y. Xia W. Xu R. Chen Y. (2021). Modified PMA-qPCR method for rapid quantification of viable *Lactobacillus spp.* in fermented dairy products. Food Anal. Methods 14, 1908–1918. doi: 10.1007/s12161-021-02022-3

[ref31] Zielińska D. Ołdak A. Rzepkowska A. Zieliński K. (2018). “Enumeration and identification of probiotic bacteria in food matrices” in Handbook of food bioengineering, advances in biotechnology for food industry. eds. Holban A. M. Grumezescu A. M. (New York, NY: Academic Press), 167–196.

